# Browning of subcutaneous fat and higher surface temperature in response to phenotype selection for advanced endurance exercise performance in male DUhTP mice

**DOI:** 10.1007/s00360-016-1036-7

**Published:** 2016-09-30

**Authors:** J. Brenmoehl, D. Ohde, E. Albrecht, C. Walz, A. Tuchscherer, A. Hoeflich

**Affiliations:** 1 Institute for Genome Biology, Leibniz-Institute for Farm Animal Biology (FBN), Wilhelm-Stahl-Allee 2, 18196 Dummerstorf, Germany; 2 Institute for Muscle Biology and Growth, Leibniz-Institute for Farm Animal Biology (FBN), Wilhelm-Stahl-Allee 2, 18196 Dummerstorf, Germany; 3 Institute for Biometry and Genetics, Leibniz-Institute for Farm Animal Biology (FBN), Wilhelm-Stahl-Allee 2, 18196 Dummerstorf, Germany

**Keywords:** Selection, Browning, Irisin, Subcutaneous fat, Uncoupling protein

## Abstract

For the assessment of genetic or conditional factors of fat cell browning, novel and polygenic animal models are required. Therefore, the long-term selected polygenic mouse line DUhTP originally established in Dummerstorf for high treadmill performance is used. DUhTP mice are characterized by increased fat accumulation in the sedentary condition and elevated fat mobilization during mild voluntary physical activity. In the present study, the phenotype of fat cell browning of subcutaneous fat and a potential effect on oral glucose tolerance, an indicator of metabolic health, were addressed in DUhTP mice. Analysis of peripheral fat pads revealed increased brite (brown-in-white) subcutaneous adipose tissues and in subcutaneous fat from DUhTP mice higher levels of irisin and different markers of fat cell browning like T-box transcription factor (Tbx1), PPARα, and uncoupling protein (UCP1) (*P* < 0.05) when compared to unselected controls. UCP1 was further increased in subcutaneous fat from DUhTP mice in response to mild exercise (fourfold, *P* < 0.05). In addition, surface temperature of DUhTP mice was increased when compared to controls indicating a physiological effect of increased UCP1 expression. The present study suggests that DUhTP mice exhibit different markers of mitochondrial biogenesis and fat browning without external stimuli. At an age of 43 days, sedentary DUhTP mice have improved metabolic health as judged from lower levels of blood glucose after an oral glucose tolerance test. Consequently, the non-inbred mouse model DUhTP represents a novel model for the identification of fat cell browning mechanisms in white adipose tissues.

## Introduction

The Dummerstorf marathon mouse model DUhTP has been selected over 90 generations for high treadmill performance (Falkenberg et al. 2000) and thus represents a unique model for the study of energy metabolism. In the liver of DUhTP mice increased lipid synthesis has been postulated based on the analysis of hepatic metabolome by current mass spectrometry (Brenmoehl et al. 2013). In the sedentary condition, marathon mice accumulate high amounts of body fat in external depots indicating physiological relevance of lipids for superior endurance exercise performance in DUhTP mice. In fact, recent work revealed that external fat mass in DUhTP mice is highly responsive to physical activity because voluntary exercise in running wheels completely abolished the obese phenotype of DUhTP mice (Brenmoehl et al. 2015). In subcutaneous fat from DUhTP mice, PGC1-α and mitochondrial DNA content were increased and it was speculated that fat cell browning may occur in subcutaneous white adipocytes (Brenmoehl et al. 2015). It is known that brite adipocytes, converted from white adipocytes, exhibit properties of brown adipocytes like expression of UCP1 and a proposed mechanism for the induction of browning in WAT may occur by irisin derived from muscle after contraction (Bostrom et al. 2012) or from the fat independent of physical exercise (Roca-Rivada et al. 2013). A number of studies has demonstrated that the addition of exogenous irisin or irisin precursors (FNDC5) induced browning of white fat cells both in rodent (Zhang et al. 2014; Bostrom et al. 2012) and in human white fat cells (Zhang et al. 2016; Huh et al. 2014; Lee et al. 2014). Fat cell browning in response to irisin treatment further induced cellular thermogenesis in mature human white fat cells (Zhang et al. 2016). Because irisin may represent an early marker of fat cell browning and thermogenesis, it may have the potential as a novel anti-obesity drug (Bostrom et al. 2012) or in a more general view could be seen as an attractive tool for the manipulation of energy metabolism in vivo.

Interestingly, after acute exercise, higher levels of irisin have been found in muscle extracts and in serum of marathon mice (DUhTP) long-term selected for endurance exercise (Brenmoehl et al. 2014) supporting the idea that irisin may act as an endogenous effector of energy metabolism.

The aim of the present work is to determine if the marathon mouse model DUhTP is a natural model for the analysis of adipose browning in the presence of elevated FNDC5/irisin expression. To accomplish this, we measured FNDC5/irisin expression and content of selected tissues, UCP1 content, and skin temperature as a surrogate for core temperature both in DUhTP mice and in unselected controls.

## Materials and methods

### Animals

All in vivo experiments were performed in accordance with national and international guidelines and were approved by our internal institutional review board. In this study, on one hand, a non-inbred mouse line that has been generated by selection over 90 generations for high treadmill performance (DUhTP) (Falkenberg et al. 2000) and on the other control mice (DUC) that had been generated from the identical base population without phenotype selection was used (Dietl et al. 2004). The males were housed under controlled environmental conditions in a semi-barrier system with a 12-h light–12-h dark cycle (room temperature = 22.5 ± 0.2 °C, humidity = 50–60 %) as described in (Brenmoehl et al. 2013). At an age of 49 days, DUhTP mice and controls were kept either in home cages with running wheels (RW) (*d* = 33.4 cm; Tecniplast, Hohenpeißenberg, Germany) to assess physical activity over a period of 3 weeks (*n* = 10 per group) or in cages without RW as controls (*n* = 10 per group). At 70 days of age, mice were fasted overnight and killed by decapitation to collect serum samples. Tissues were weighted (summary in Table 1), snap-frozen in liquid nitrogen, and stored at −70 °C for subsequent analysis. In parallel, mice (*n* = 9 per group) were analyzed under temperature conditions of husbandry at 22 °C for surface temperature using an infrared camera (Testo 881; Testo AG, Lenzkirch, Germany).

**Table 1:** Body and tissue weights of male mice long-term selected for high treadmill performance (DUhTP) and unselected controls (DUC) at an age of 70 days as published before (Brenmoehl et al. 2015)

	*n*	DUhTP	DUhTP-RW	*P*	DUC	DUC-RW	*P*	*P* (DUhTP vs DUC)	*P* (DUhTP-RW vs DUC-RW)
Body mass (g)	10	32.18 ± 1.02	31.25 ± 0.87	0.4925	35.18 ± 0.67	34.65 ± 0.79	0.6125	**0.0191**	**0.0066**
Lean body mass (g)	10	20.89 ± 1.76	20.5 ± 1.78	0.7529	23.56 ± 1.44	23.45 ± 1.61	0.8702	**0.0025**	**0.0007**
Body length (cm)	10	10.04 ± 0.12	9.93 ± 0.11	0.4907	10.44 ± 0.10	10.42 ± 0.09	0.8845	**0.0135**	**0.0014**
Musculus rectus femoris (g)	10	0.37 ± 0.01	0.38 ± 0.01	0.2969	0.39 ± 0.01	0.36 ± 0.01	0.0795	0.1378	0.1767
Musculus rectus femoris (%)	10	0.0114 ± 0.00	0.0122 ± 0.00	**0.0372**	0.0111 ± 0.00	0.0104 ± 0.00	0.0862	0.3829	**0.0004**
Liver mass (g)	10	1.81 ± 0.07	1.76 ± 0.08	0.6301	1.94 ± 0.06	1.82 ± 0.06	0.1513	0.1571	0.5192
Liver mass (%)	10	0.0561 ± 0.00	0.0561 ± 0.00	0.9855	0.0552 ± 0.00	0.0525 ± 0.00	0.1388	0.5363	0.0758
Subcutaneous fat (g)	10	0.36 ± 0.03	0.22 ± 0.03	**0.0012**	0.20 ± 0.01	0.18 ± 0.02	0.4910	**0.00003**	0.2448
Subcutaneous fat (%)	10	0.0112 ± 0.00	0.0065 ± 0.00	**0.0002**	0.0056 ± 0.00	0.0053 ± 0.00	0.6095	**0.00001**	0.1495
Epidymal fat (g)	10	0.33 ± 0.03	0.23 ± 0.03	**0.0145**	0.28 ± 0.03	0.20 ± 0.02	**0.0183**	0.2122	0.2718
Epidymal fat (%)	10	0.0102 ± 0.00	169 ± 0.00	**0.0025**	179 ± 0.00	156 ± 0.00	**0.0195**	**0.0320**	0.1383
Perinephric fat (g)	10	0.16 ± 0.02	0.08 ± 0.01	**0.0020**	0.09 ± 0.01	0.06 ± 0.01	**0.0086**	**0.0035**	0.2327
Perinephric fat (%)	10	0.0049 ± 0.00	0.0021 ± 0.00	**0.0002**	0.0025 ± 0.00	0.0017 ± 0.00	**0.0083**	**0.0003**	0.2122
Brown fat (g)	10	0.09 ± 0.01	0.07 ± 0.00	**0.0191**	0.06 ± 0.00	0.06 ± 0.00	0.7512	**0.0006**	0.1189
Brown fat (%)	10	0.0027 ± 0.00	0.0022 ± 0.00	**0.0361**	0.0017 ± 0.00	0.0017 ± 0.00	0.8656	**0.00005**	**0.0103**

Mice were kept in cages with running wheels (RW) or without. Total body lean mass was analyzed by dual energy X-ray absorptiometry. Values are presented as mean ± SE. Significant group effects (*P*) are indicated in bold letters (%: normalized by body weight)

### Oral glucose tolerance test

Oral glucose tolerance tests (oGTT) were performed in mice at an age of 43 and 71 days, essentially as described before (Renne et al. 2013). In brief, glucose was applied to overnight fasted mice (43 days: *n* = 20; 71 days: *n* > 5 per group) in an oral dose of 1 g glucose/kg body weight dissolved in tap water and concentrations of blood glucose were assessed before and 10, 30, 60, and 120 min after the oral glucose bolus. Glucose concentrations were analyzed using a glucometer (Roche, Penzberg, Germany).

### Quantitative real-time PCR

Expression of different mRNA transcripts in subcutaneous fat samples (n = 7) was analyzed in triplicates as described previously (Brenmoehl et al. 2014, 2015). Primers used are mTbx1-forw: ggcaggcagacgaatgttc, mTbx1-rev: ttgtcatctacgggcacaaag, UCP1-forw: ggcctctacgactcagtcca, UCP1-rev: taagccggctgagatcttgt, Tcf21-forw: cattcacccagtcaacctga, Tcf21-rev: ttccttcaggtcattctctgg, Fndc5-forw: caacgagcccaataacaaca, Fndc5-rev: agaaggtcctctcgcattctc. Different housekeeping genes (Brenmoehl et al. 2015) were tested to identify the HKG with comparable Crossing point (Cp)-values (Cp_Rplp2_: DUhTP: 24.45 ± 1.17; DUC: 25.80 ± 1.03) to normalize expression of *Tbx1* and *Ucp1*.

### Immunoblotting

Western immunoblotting was performed as described previously (Brenmoehl et al. 2014). Equal loading of the gels and proper transfer of the proteins to the membranes were verified by Coomassie Blue staining according to standard procedures (Taylor et al. 2013). Expression of UCP1 (sc-6529, Santa Cruz, Heidelberg, Germany), irisin (A00170-01-100; Bio Trend, Cologne, Germany), and FNDC5 (AP8746b; Abgent, San Diego, CA, USA) in all ten animals per group were studied by Western immunoblotting. Western immunoblots were repeated at least three times. Thereby, samples were determined in different order to avoid position effects while blotting.

### Immunohistochemical staining in adipose tissue

In addition to standard hematoxylin/eosin (H/E) staining, immunohistochemistry was applied for UCP1 and C/EBPβ detection on 12 µm thick frozen tissue sections (2–3 randomly selected mice per group) using antibodies against either UCP1 (1:100, sc-6529, Santa Cruz) for 1 h at room temperature or C/EBPβ (1:100, sc-150, Santa Cruz) for 2 h at room temperature in a humidity chamber similar to previously described procedure (Albrecht et al. 2015a). Specific binding was detected with fluorescence-labeled secondary antibodies (MFP 488 rabbit anti- goat IgG for UCP1 and MFP 590 goat anti-rabbit IgG for C/EBPβ, MoBiTec, Goettingen, Germany). All sections were counterstained with 1 µg/ml Hoechst 33258 (Sigma-Aldrich, Munich, Germany). Immunofluorescence was visualized with a Nikon Microphot SA fluorescence microscope (Nikon Instruments Europe B.V., The Netherlands) and an image analysis system equipped with CELL^F image analysis software and a CCD-12 high resolution color camera (OSIS, Münster, Germany). The selected H/E images are representative in any case for a region of typical unilocular white adipocytes and a region of multilocular adipocytes. Fluorescence images represent a region of multilocular cells to illustrate protein expression typical for either heat producing or differentiating adipocytes. Subcutaneous fat samples were cryosectioned (25 μm thick) using a Leica CM3050 S (Leica, Bensheim, Germany) cryostat microtome. Sections were stained with hematoxylin/eosin (H/E; hematoxylin: Dako, Glostrup, DK; eosin: Chroma Gesellschaft, Münster, Germany) and embedded with Roti-Histokit (Roth, Karlsruhe, Germany). Adipocyte size was measured using the interactive measurement module of an image analysis system equipped with an Olympus BX43 microscope (Olympus, Hamburg, Germany), an UC30 color camera (Olympus) and CELL^D image analysis software (OSIS, Munster, Germany). About 300 adipocytes per sample were randomly selected and measured, after following the contour using the interpolating polygon function.

### Statistical analysis

The data analysis was performed using SAS software (Version 9.4 for Windows, SAS Institute Inc., Cary, NC, USA). Descriptive statistics and tests for normality were calculated with the UNIVARIATE procedure of Base SAS software (SAS Institute Inc. 2013. Base SAS^®^ 9.4 Procedures Guide, Second Edition. Cary, NC: SAS Institute Inc.). Data considered approximately normal were analyzed by ANOVA using the GLIMMIX procedure of SAS/STAT software (SAS Institute Inc. 2013. SAS/STAT^®^ 13.1 User’s Guide. Cary, NC: SAS Institute Inc.). The ANOVA model for the 70 days data included the fixed factors line (levels: DUC, DUhTP), group (levels: co, RW), and the interaction line × group. Homogeneity of covariance parameters across groups was tested for all ANOVA models. In addition, least-squares means (LSM) and their standard errors (SE) were computed for each fixed effect in the models, and all pairwise differences of LS-means were tested by the Tukey–Kramer procedure. Effects and differences were considered significant if *P* < 0.05. Glucose levels in response to oral glucose application were further analyzed by repeated measurements ANOVA provided by the BASE SAS software package.

## Results

### Irisin in subcutaneous adipose tissue

**Fig. 1:**
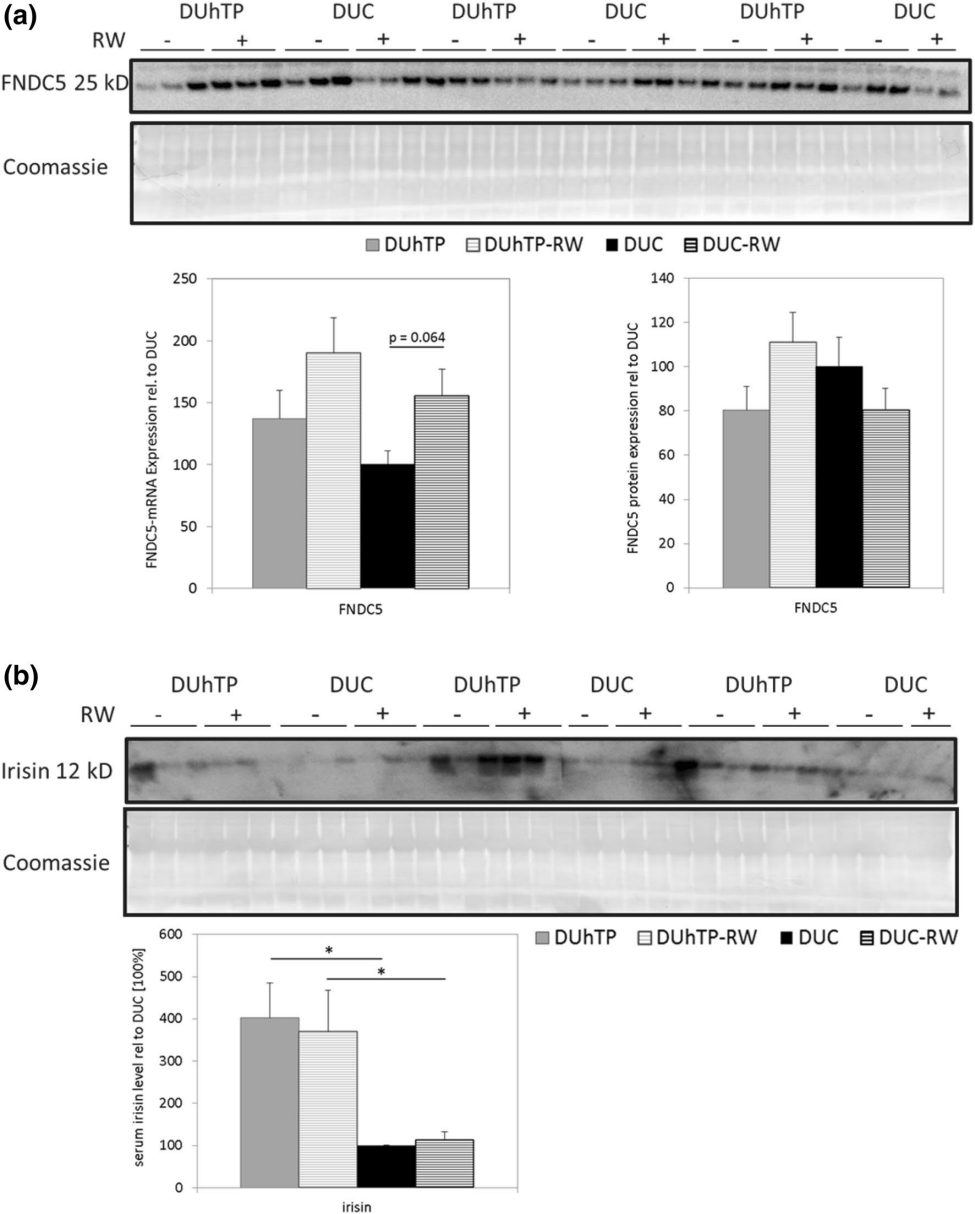

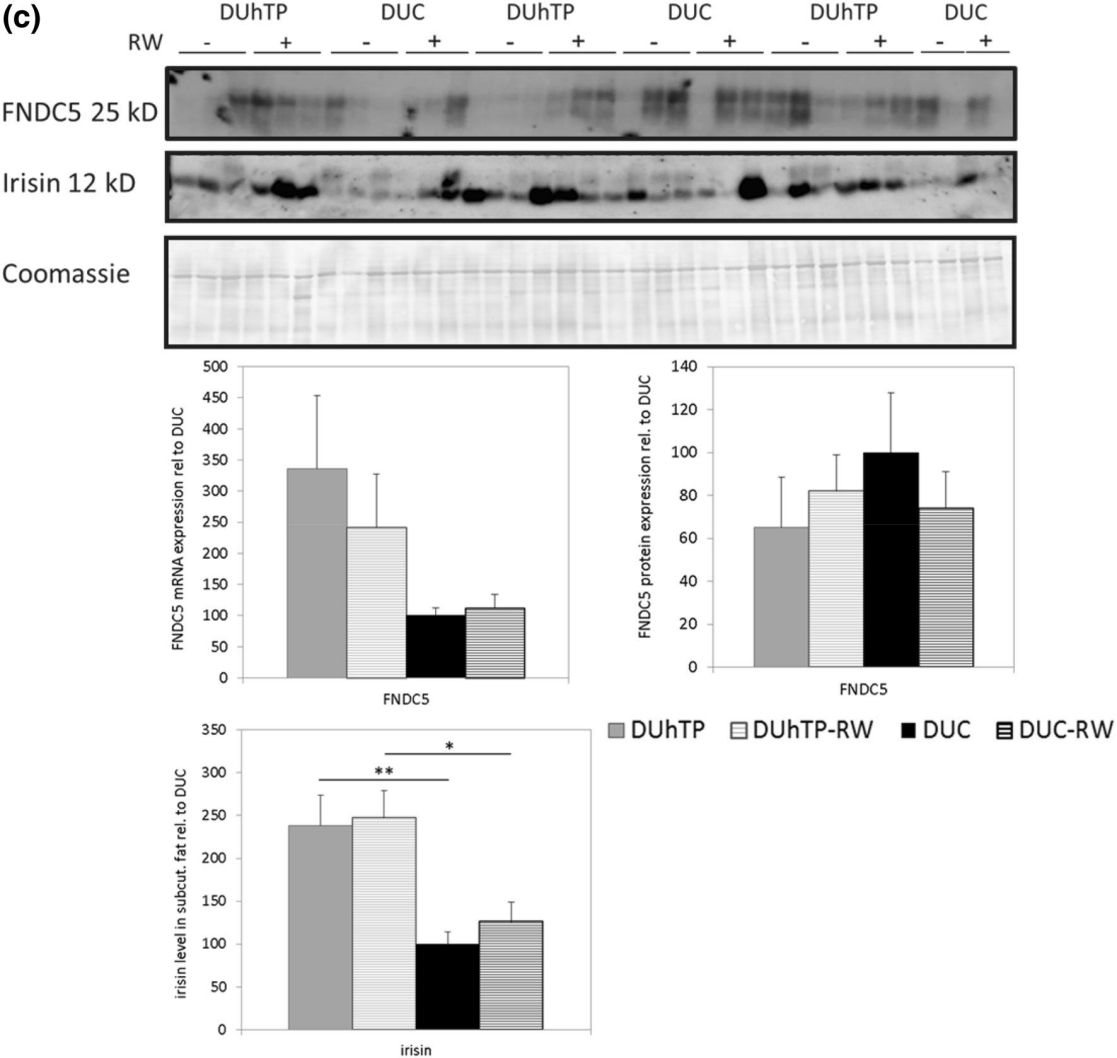
Effect of voluntary physical activity (RW) on levels of **a** FNDC5 in muscles, **b** irisin in serum and **c** FNDC5 and irisin in subcutaneous adipose tissue from DUhTP and DUC mice. Analysis of FNDC5-mRNA expression was performed by quantitative real-time PCR and normalized to expression of HKG Rplp2. Protein analysis was performed by Western immunoblotting. Protein expression was quantified by densitometry and normalized for the Coomassie blue signal. All data are expressed in relation to the expression level of sedentary unselected controls (DUC) with no access to RW. Values are mean ± SE; *P < 0.05, **P < 0.005

Previously, expression of FNDC5 protein in muscles and abundance of irisin in serum of DUhTP mice was demonstrated (Brenmoehl et al. 2014). Now expression of irisin and FNDC5 was analyzed in active and sedentary DUhTP and control mice. Both mouse lines voluntarily used running wheels present in their home cages to a similar extent (DUhTP: 3935 ± 1719; DUC 3913 ± 1181 m/day). A comparison of FNDC5 and irisin between both sedentary mouse lines revealed abundant but unchanged levels of muscular FNDC5 (Fig. 1a) but higher levels of irisin (Fig. 1b) in serum of DUhTP mice (~fourfold, *P* < 0.05). Physical activity had no influence on FNDC5 or irisin levels. Indeed, very high concentrations of irisin were detected in subcutaneous adipose tissue of DUhTP mice (Fig. 1c). In contrast to controls, sedentary and active DUhTP mice showed over twofold increased irisin levels; FNDC5 protein was barely detectable in subcutaneous adipose tissue and showed no clear differences.

### Elevated browning in subcutaneous fat from DUhTP mice

**Fig. 2:**
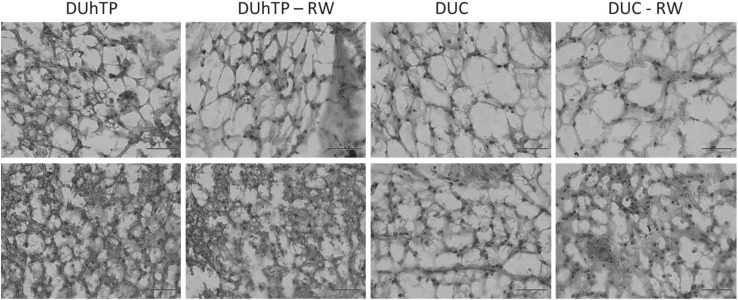
Hematoxylin/Eosin staining of subcutaneous adipose tissue from sedentary and physical active (−RW) male DUhTP and DUC mice, respectively. *Upper set* of *pictures* displays areas of white adipocytes and *lower pictures* show area with clusters of brite adipocytes. *Scale bar* 50 µm

**Fig. 3:**
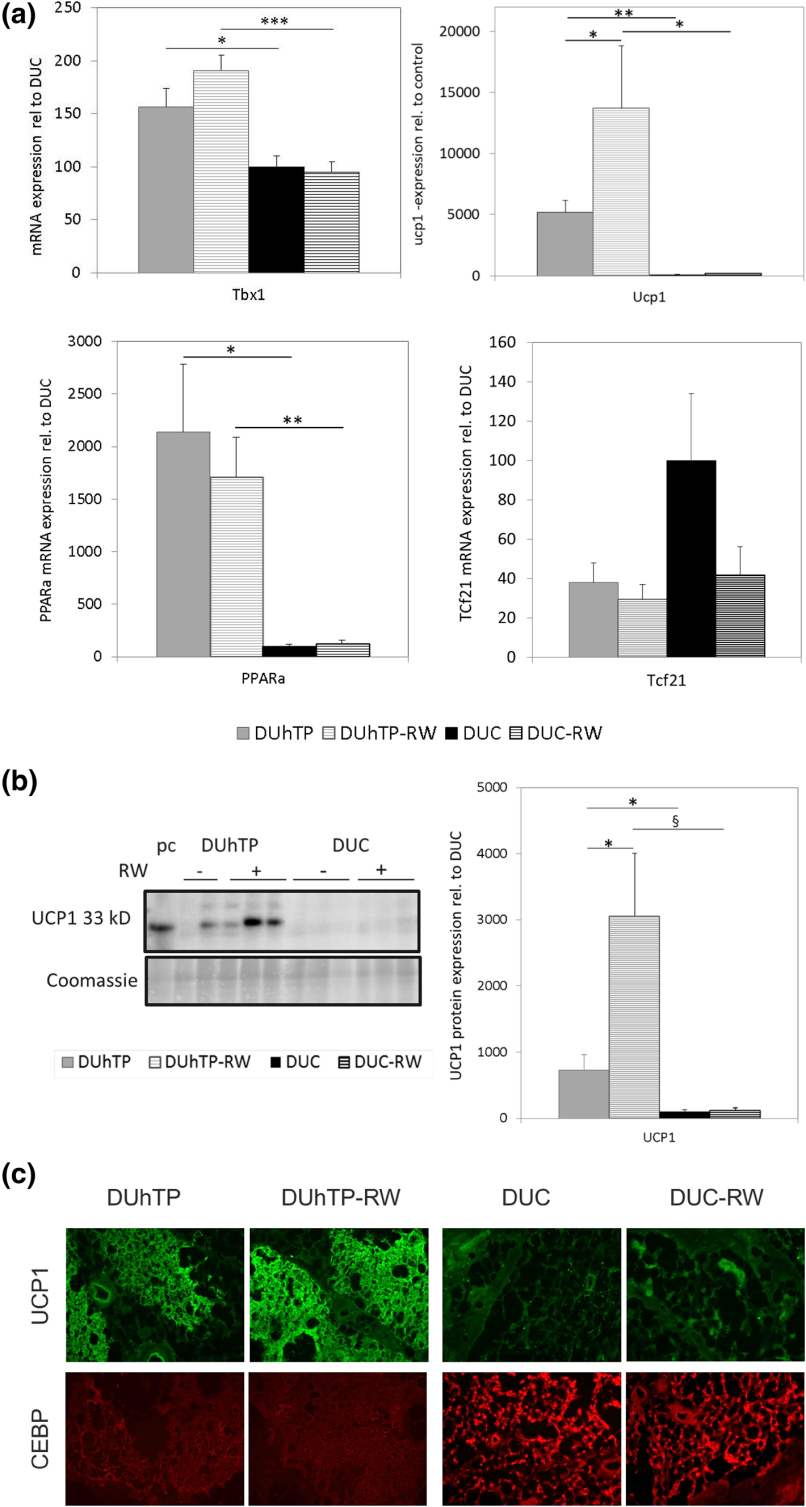
Effect of voluntary physical activity (RW) on mRNA expression (**a**) of brite adipose tissue marker Tbx1 (*upper left panel*), brown adipose tissue marker UCP1 (*upper right panel*) as well as PPARα (*lower left panel*) and white adipose tissue marker Tcf21 (*lower right panel*) in subcutaneous fat tissue of 10-week male DUhTP and DUC mice (*n* = 7 per group). Analysis of mRNA expression was performed by quantitative real-time PCR and normalized to expression of HKG Rplp2. The analysis was performed by Western immunoblotting. Protein expression of UCP1 (**b**) was quantified by densitometry and normalized for the Coomassie blue signal. UCP1 (*green*) and C/EBPβ protein (*red*) were also analyzed by immunohistochemistry (**c**) in subcutaneous adipose tissue of 10-week male DUhTP and DUC mice. Cryosections of running wheel (RW) exercised and sedentary mice were immunostained with anti-UCP1 and C/EBPβ primary antibodies and MFP 488 rabbit anti- goat IgG secondary antibody for UCP1 and MFP 590 goat anti-rabbit IgG for C/EBPβ. Data are presented as mean ± SE and are expressed relative to the expression level of control mice from line DUC. Significant differences are indicated: *P < 0.05, **P < 0.005; ***P < 0.0005; ^§^ *P *< 0.01 (C/EBPβ CCAAT enhancer-binding protein beta, Tbx1 T-box transcription factor 1, UCP1 uncoupling protein 1, PPARα, pc positive control) (color figure online)

Subcutaneous fat dissected from DUhTP mice of both treatment groups appeared darker when compared to unselected control mice, which suggested differential browning in DUhTP and DUC mice. Histological examination revealed clustered formation of smaller multilocular adipocytes (brite fat cell) and larger unilocular adipocytes (white fat cell) in tissue sections from subcutaneous fat of both mouse lines at an age of 10 weeks (Fig. 2). Clustered appearance of white and brite fat cells was also observed in 90-day-old mice of both genetic groups. Examination of the tissue sections indicated larger areas covered by brite fat cells in DUhTP versus DUC mice. In white adipocytes, histological parameters (area, diameter, and Feret diameter) were unaffected by genetic group of voluntary physical activity (data not shown). To describe the differential phenotype of fat cell browning, biomarkers of brown, white, and brite fat cells were assessed in subcutaneous fat (Fig. 3a). Tbx1 mRNA expression was significantly increased in DUhTP mice (56 %; *P* < 0.05) compared to unselected controls. Physical activity in DUhTP had no significant effect on mRNA expression of the brite adipose tissue marker Tbx1 (22 %, n.s.). As expected, white adipose tissue marker transcription factor 21 (Tcf21) was barely expressed in sedentary DUhTP mice when compared to control mice but the differences between both mouse lines reached no statistical significance because of high individual variability. Transcription factor PPARα was also elevated in the subcutaneous fat of active and sedentary DUhTP mice in contrast to control animals (17-fold; *P* < 0.005 and 21-fold; *P* < 0.05, respectively). Downstream expression of BAT-marker Ucp1 was increased in subcutaneous fat of DUhTP mice as well (50-fold; *P* < 0.005). In response to activity, expression of Ucp1 further increased threefold (*P* < 0.05). In controls, expression of Tbx1, PPARα, and Ucp1 were unaffected by physical activity.

Higher expression of UCP1 in DUhTP mice in subcutaneous fat was present also on the protein level as demonstrated by Western immunoblotting (Fig. 3b). Compared to sedentary DUC mice, UCP1 was 7.3-fold increased in sedentary DUhTP mice. Moderate voluntary activity for 3 weeks resulted in a further 4.2-fold higher protein expression in subcutaneous fat of DUhTP mice when compared to non-exercised controls. Higher protein levels of UCP1 were further demonstrated by immunohistochemistry. Cryostat sections of subcutaneous adipose tissue of sedentary and physical active DUhTP mice displayed areas of multilocular adipocytes. These areas stained massively for UCP1, while in comparable sections of control mice, only weak UCP1 protein expression was detectable (Fig. 3c). Multilocular adipocytes in control mice could be confirmed as differentiating adipocytes with positive staining for CCAAT/enhancer-binding protein beta (C/EBPβ). Only few C/EBPβ-positive adipocytes were observed in UCP1-stained regions of DUhTP mice.

**Fig. 4:**
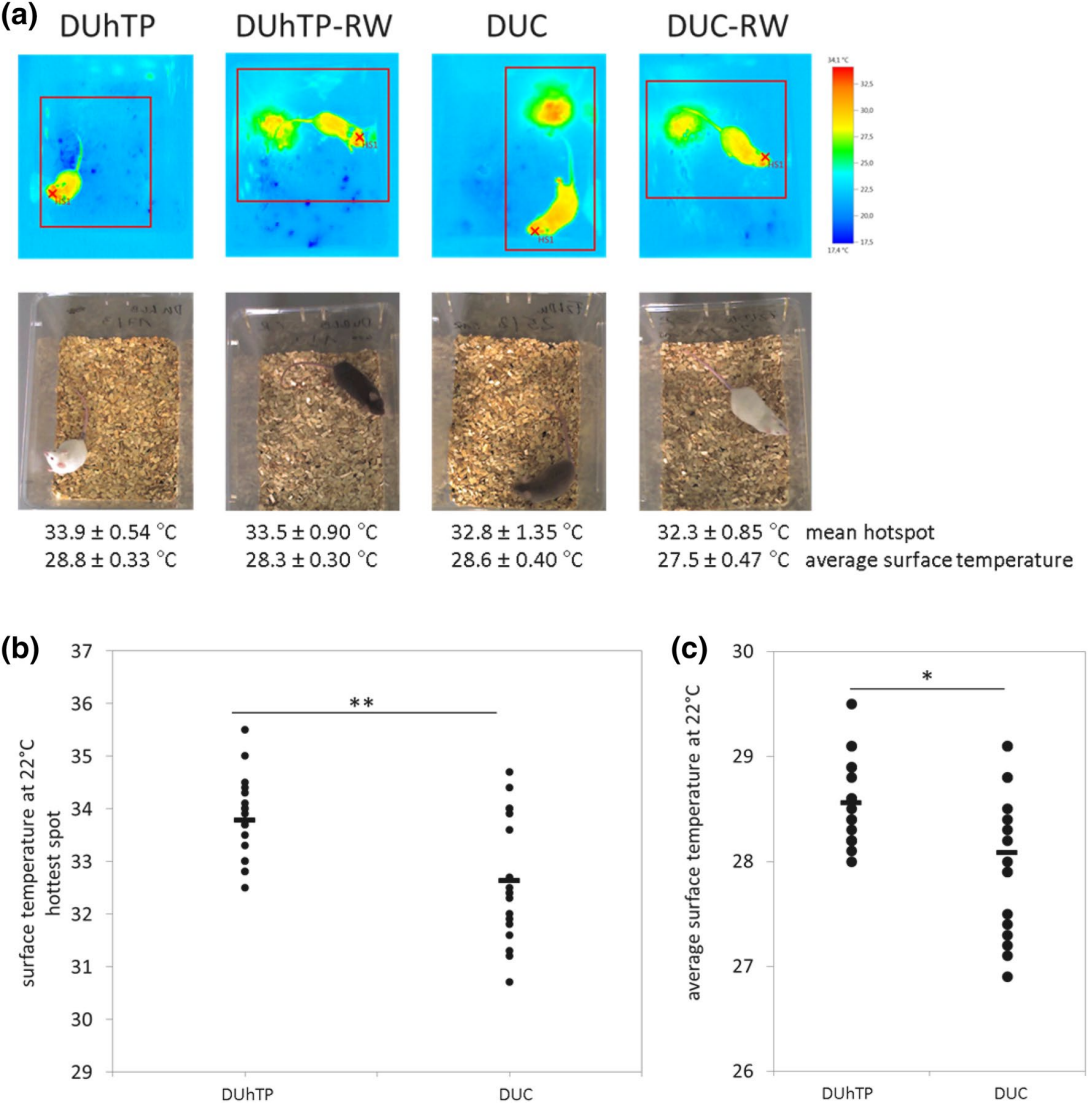
Analysis of surface temperature of physical active and sedentary DUhTP and DUC mice (*n* = 9 per group). Mice were photographed at 22 °C using infrared technology (**a**). *Normal photos* were taken in parallel. Testo software determined the hottest spot (**b**) and the average surface temperature (**c**) of the mouse. The displayed mouse with according surface temperature is representable for each group-average. Significant differences are indicated: *P < 0.05; **P < 0.005

UCP1 is known to uncouple ATP production leading to enhanced energy production, allowing small animals to better tolerate cold (Himms-Hagen 1970). As an indirect and noninvasive method to determine UCP1 activity, heat production indicated by change in surface temperature was measured in both mouse lines using an infrared camera. It was asked if the increased UCP1 expression in subcutaneous fat correlates with an elevated surface temperature at 22 °C room temperature. In fact, DUhTP mice showed a significantly higher surface temperature (+1 °C; *P* < 0.005) than control mice (Fig. 4), but differences between active and sedentary littermates were not found.

### Better oral glucose tolerance in DUhTP mice

**Fig. 5:**
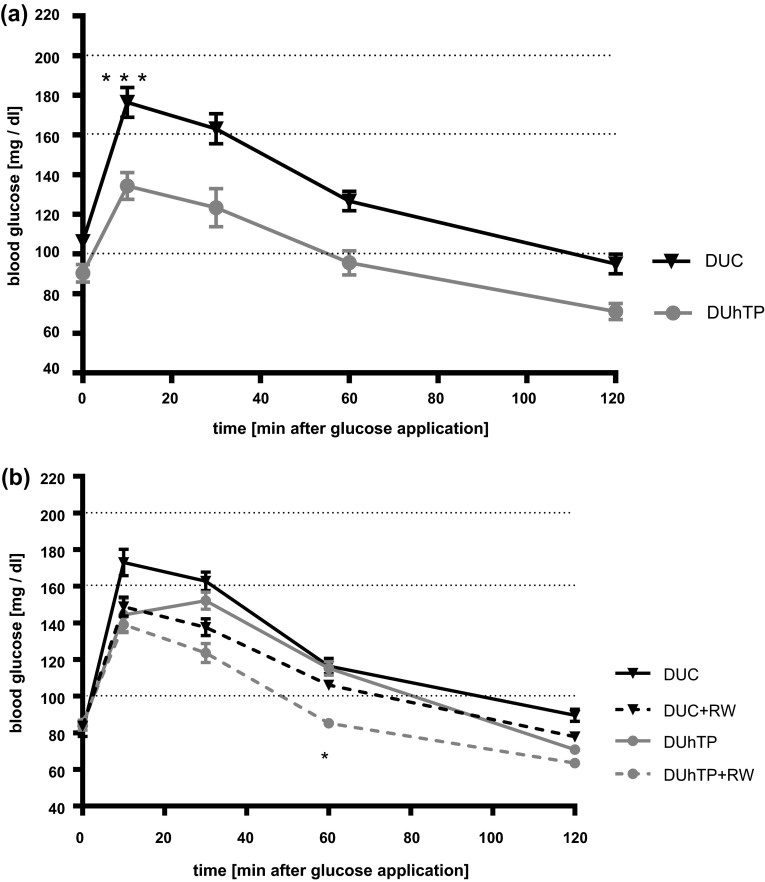
Oral glucose tolerance tests (oGTT) in male DUhTP and DUC mice under standard chow at an age of 43 days (**a**; *n* = 20) and at an age of 71 days (**b**; *n* > 5). At an age of 43 days for all pairwise comparisons, significant differences were found between DUhTP mice and controls fed standard diet (*P* < 0.01). At an age of 71 days in the presence of running wheels (RW), reduced blood glucose were measured in DUhTP mice if compared to other groups (*P < 0.05)

At an age of 43 days, male DUhTP mice had lower blood glucose levels before and after oral application of glucose (Fig. 5a) at all time points assessed (*P* < 0.01). Lower blood glucose levels were not found in elder DUhTP mice at an age of 71 days (Fig. 5b). However, voluntary physical activity over a period of 3 weeks in DUhTP mice significantly reduced blood glucose (*P* < 0.05). The areas under curve of oral glucose levels after oral glucose tolerance tests in DUhTP mice and unselected controls were statistically not significantly different (data not shown). However, repeated measurement ANOVA revealed a significant interaction between the time of glucose testing and mouse line (*P* < 0.05).

## Discussion

Irisin has been identified as an effector of fat cell browning and thermogenesis by the induction of UCP1 (Bostrom et al. 2012). In addition, irisin effects on carbohydrate and lipid metabolism also have been provided for the liver (Mo et al. 2016) suggesting broader effects on energy metabolism. Our marathon mouse model DUhTP, established by long-term selection for high treadmill performance, is characterized by increased hepatic lipogenesis on one hand and peripheral obesity on the other, if compared to unselected control mice (DUC) (Brenmoehl et al. 2013). For the sake of clarity, we included Table 1 in our manuscript providing published data on increased fat accretion in DUhTP mice (Brenmoehl et al. 2015). Notably, in DUhTP also irisin concentrations were found being increased in skeletal muscle and plasma (Brenmoehl et al. 2014). To establish DUhTP mice as an in vivo-relevant and polygenic model of irisin actions, we tested current hypotheses in DUhTP mice. We therefore assessed known effects of irisin in subcutaneous fat and investigated fat cell morphology, browning, UCP1 expression, and thermogenesis in our experimental system. Finally we asked if higher irisin expression in DUhTP also correlated with improved metabolic health.

Adipose tissue of DUhTP mice showed more multilocular adipocytes than DUC mice, with no obvious effect on white adipocyte histology. Real-time PCR and immunohistochemical analyses revealed lower expression of markers associated with white (C/EBP; Tcf21), brite (Tbx1) and brown adipose tissue (UCP1, PPARα) (Escher et al. 2001; Wu et al. 2012) arguing for elevated abundance of brite adipocytes in this fat depot of DUhTP mice already under sedentary conditions. In response to 3 weeks of voluntary exercise, UCP1-expression was further increased in DUhTP mice, whereas in controls, no changes were detectable. High UCP1 abundance in DUhTP and weak expression in control mice were confirmed by immunohistochemistry. Especially UCP1, a known regulator of BAT-dependent thermogenesis (Argyropoulos and Harper 2002) with low-level expression in WAT (Wu et al. 2012), indicates the presence of brite cells in subcutaneous fat. Fat cell browning or enhancement of mitochondrial biogenesis in WAT, respectively, is part of the thermogenic program and is induced and activated by the transcriptional regulator PGC1-α leading to increased expression of FNDC5 and after cleavage of FNDC5 higher circulating levels of irisin (Bostrom et al. 2012; Handschin and Spiegelman 2008). Recently, a study on PGC1-α in muscles of DUhTP mice after endurance exercise on a treadmill provided increased expression of PGC1-α isoforms 1, 3, and 4 on mRNA level (Brenmoehl et al. 2014). Voluntarily exercised mice only showed alterations of PGC1-α isoform 1 mRNA when compared to sedentary littermates (Brenmoehl et al. 2014). These observations nicely agree with those of Ruas et al., who linked PGC1-α isoform 1 mRNA to endurance performance but isoform 4 to resistance training (Ruas et al. 2012). FNDC5 is highly abundant in muscle, rectum, heart, and pericardium but present also in fat, brain, kidney, and liver, however, with lower abundance (Huh et al. 2012). Here, we assessed FNDC5 and irisin in muscle, serum, and subcutaneous fat of both mouse lines. The existence of FNDC5 in muscle and irisin in serum of DUhTP mice had originally been described using an antibody that was able to detect the irisin band of 12 kDa by Western immunoblotting (Brenmoehl et al. 2014). Also in the present work, this antibody was used. As far as we know, this antiserum is the only one that recognizes recombinant irisin with its correct molecular weight of 12 kDa (Albrecht et al. 2015b). Currently, this antiserum is not available because production and distribution have been halted. As discussed recently, different antibodies or antisera used for western immunoblotting or in ELISA studies revealed a number of bands but none of them corresponded to the correct size of irisin at ~12 kDa (Albrecht et al. 2015b). Instead only FNDC5 was identified by different antisera assessed in the study of Albrecht and coauthors. Barja-Fernandez et al. (2016) could detect a 15 kDa band stained by an irisin antibody that contains indeed no specific peptide signature for irisin. In the present study, higher levels of irisin were observed in serum and subcutaneous adipose tissue of DUhTP mice when compared with controls. However, expression of FNDC5 did not vary between both genotypes in muscle and in subcutaneous fat. Low abundance of FNDC5 protein in subcutaneous fat in contrast to muscular FNDC5 protein is also observed in human studies. Thus, higher local irisin concentrations in subcutaneous fat may be due to transport of muscular-derived irisin to adipose tissue via the blood stream or to enhanced release from other tissues like liver, heart, rectum or brain (Huh et al. 2012). We further cannot exclude the possibility that increased irisin levels arise from enhanced proteolytic cleavage of FNDC5. In response to voluntary activity, changes in FNDC5 expression and irisin levels were not observed in serum or subcutaneous fat when compared to sedentary littermates. Alterations of irisin levels independent of physical activity were only detectable in serum and muscle of our mouse line DUhTP passing a submaximal running test on a treadmill (Brenmoehl et al. 2014). It seems that voluntary physical activity has no influence on irisin level in serum and subcutaneous fat in either mouse line but browning of subcutaneous fat as a potential effect of irisin was increased after 3 weeks of moderate exercise.

In cultured skeletal muscle cells, irisin administration results in increased oxidative metabolism and elevated energy expenditure by induction of metabolic genes like PGC1-α, Tfam, GLUT4 or UCP3 (Vaughan et al. 2014). In primary cultured rat adipocytes from subcutaneous fat pads a direct administration of recombinant irisin significantly increased gene expression of UCP1 and PGC1-α (Zhang et al. 2014). Daily treatment of normal and obese mice with injection of recombinant irisin for 2 weeks led to elevated levels of Ucp1, Pgc1-α, and Pparα mRNA in subcutaneous fat (Zhang et al. 2014). In mice that were injected with FNDC5-expressing adenovirus, subcutaneous fat pads expressed high levels of Pgc1-α mRNA as well as UCP1 mRNA and protein (Bostrom et al. 2012).

Otherwise, irisin is thought to increase expression of PGC1-α in adipose tissue (Bostrom et al. 2012). We could detect high levels of transcription cofactor PGC1-α mRNA and protein in subcutaneous fat of DUhTP mice compared to unselected controls (Brenmoehl et al. 2015). Additional voluntary exercise resulted in an increase of PGC1-α protein levels. The requirement of PGC1-α to induce acute exercise-mediated Ucp1 mRNA expression in WAT was demonstrated by Ringholm et al. (Ringholm et al. 2013). They found in PGC1-α KO mice no changes in expression of UCP1 protein in response to exercise training. PGC1-α acts in muscle and adipose tissue as an inducer of mitochondrial biogenesis leading to elevated mitochondrial mass, proteins, and capacity (Lin et al. 2002; Puigserver and Spiegelman 2003; Olesen et al. 2010). DUhTP mice also express high amounts of Tfam, complex I subunits ND1 and NDUFA9, and SIRT3. Voluntary activity increased nuclear encoded proteins NDUFA9 and SIRT3 (Brenmoehl et al. 2015), thus implicating higher mitochondrial biogenesis in subcutaneous fat from DUhTP mice associated with higher potential of energy supply.

Detection of substantial irisin levels in subcutaneous fat of DUhTP mice further inspired characterization of adipose tissue of sedentary DUhTP and DUC mice. PPARα, induced by irisin (Huh et al. 2014), is known to induce transcription of UCP1 in adipose tissue (Puigserver 2005) and correlates with the levels of UCP1 (Xue et al. 2005). In the present investigations, high expression levels of both PPARα and UCP1 in DUhTP mice were identified. Interestingly, positive correlations between Pparα and Ucp1 mRNA expression (*R* ^2^ = 0.73) and PPARα mRNA and UCP1 protein expression (*R* ^2^ = 0.61) were only identified following voluntary activity in DUhTP mice (data not shown). In DUhTP mice, moderate voluntary activity further increased UCP1 levels, which, however, were not reflected by alterations of local irisin concentrations. Thus, we cannot exclude that other mechanisms may contribute to activity-related increases of UCP1 in subcutaneous fat. Precisely, because mitochondrial protein UCP1 uncouples the electron transport chain from energy production, resulting in heat release (Aquila et al. 1985; Argyropoulos and Harper 2002), the surface temperature of both mouse lines was investigated at retained husbandry temperature of 22 °C without any cold stress. The surface temperature was increased by 1 °C in DUhTP mice compared to controls. Noninvasive detection of surface temperature by infrared camera identified brown adipose tissue as “hottest spot” of heat production. From the present experiments, it is not clear if core temperature also is increased in DUhTP mice, which can be seen as a limitation of the present study. Subcutaneous fat seems to contribute to higher thermogenesis in DUhTP mice since UCP1 production in brown adipose tissue does not differ between both mouse lines (data not shown). Interestingly, endurance exercise in inbred mice over a period of 6 weeks produced a similar phenotype but in the muscle (Morton et al. 2013). Endurance exercised mice fed a high fat diet were characterized by higher FNDC5, PGC1-α, and UCP1 expression and fat cell browning in muscle tissues was discussed in a context of adaptive response to endurance exercise (Morton et al. 2013). While browning in muscle with mitochondrial biogenesis can be interpreted in a context of higher production of energy equivalents, browning and UCP1 production in subcutaneous fat is less clear. On one hand, the activity of UCP1 can be considered as an energy-consuming or even -wasting process. On the other hand, higher surface temperature may also increase biochemical reaction-kinetics associated with endurance performance. To support this idea, again core temperature would have to be assessed in separate studies. The contribution of UCP1 activation and increased surface temperature to physical endurance performance of DUhTP mice needs to be addressed in future studies. At an age of 43 days, DUhTP mice have improved glucose tolerance if fed a normal diet, which is in line with current concepts of metabolic health associated with brown adipose tissues (Stanford et al. 2015). With advanced age improved glucose tolerance is progressively lost in DUhTP mice. Nevertheless, voluntary physical activity maintained improved glucose tolerance at an age of 71 days in DUhTP mice. The reason for the additional increase of UCP1 expression in subcutaneous fat from DUhTP in the presence of running wheels and its specific function for metabolic health is certainly worth of a follow-up study in the future.

To summarize and conclude, the present findings indicate that subcutaneous adipose tissue in DUhTP mice is characterized by increased irisin levels and a substantial browning phenotype if compared to unselected controls. Browning of subcutaneous fat in DUhTP mice includes increased levels of Tbx1, PPARα, UCP1, and heat production and correlates with improved oral glucose tolerance at an age of 43 days. While other animal models or humans may exhibit fat cell browning and mitochondrial biogenesis particularly in subcutaneous fat for instance after long and repeated training (Stanford et al. 2015), DUhTP mice have acquired energetic and metabolic adaptations since birth and thus can display fat cell browning in sedentary conditions. Thus, this study demonstrates that the mouse model DUhTP may represent a unique polygenic model for the analysis of mechanism of fat cell browning without previous training, cold exposure or calorie restriction.
